# Characterization of White Matter Hyperintensities in Large-Scale MRI-Studies

**DOI:** 10.3389/fneur.2019.00238

**Published:** 2019-03-26

**Authors:** Benedikt M. Frey, Marvin Petersen, Carola Mayer, Maximilian Schulz, Bastian Cheng, Götz Thomalla

**Affiliations:** Department of Neurology, University Medical Center Hamburg-Eppendorf, Hamburg, Germany

**Keywords:** white matter hyperintensities, white matter lesions, systematic review, large-scale studies, white matter hyperintensity segmentation, segmentation, cerebral small vessel disease

## Abstract

**Background:** White matter hyperintensities of presumed vascular origin (WMH) are a common finding in elderly people and a growing social malady in the aging western societies. As a manifestation of cerebral small vessel disease, WMH are considered to be a vascular contributor to various sequelae such as cognitive decline, dementia, depression, stroke as well as gait and balance problems. While pathophysiology and therapeutical options remain unclear, large-scale studies have improved the understanding of WMH, particularly by quantitative assessment of WMH. In this review, we aimed to provide an overview of the characteristics, research subjects and segmentation techniques of these studies.

**Methods:** We performed a systematic review according to the PRISMA statement. One thousand one hundred and ninety-six potentially relevant articles were identified via PubMed search. Six further articles classified as relevant were added manually. After applying a catalog of exclusion criteria, remaining articles were read full-text and the following information was extracted into a standardized form: year of publication, sample size, mean age of subjects in the study, the cohort included, and segmentation details like the definition of WMH, the segmentation method, reference to methods papers as well as validation measurements.

**Results:** Our search resulted in the inclusion and full-text review of 137 articles. One hundred and thirty-four of them belonged to 37 prospective cohort studies. Median sample size was 1,030 with no increase over the covered years. Eighty studies investigated in the association of WMH and risk factors. Most of them focussed on arterial hypertension, diabetes mellitus type II and Apo E genotype and inflammatory markers. Sixty-three studies analyzed the association of WMH and secondary conditions like cognitive decline, mood disorder and brain atrophy. Studies applied various methods based on manual (3), semi-automated (57), and automated segmentation techniques (75). Only 18% of the articles referred to an explicit definition of WMH.

**Discussion:** The review yielded a large number of studies engaged in WMH research. A remarkable variety of segmentation techniques was applied, and only a minority referred to a clear definition of WMH. Most addressed topics were risk factors and secondary clinical conditions. In conclusion, WMH research is a vivid field with a need for further standardization regarding definitions and used methods.

## Introduction

Cerebrovascular disease represents a major burden on an individual as well as societal level, with growing importance in the aging western societies. Stroke as the most prominent example is the second most frequent cause of death in the world and the most frequent cause of acquired permanent disability (1). Vascular dementia represents another manifestation of cerebrovascular disease and is the second most frequent type of dementia following Alzheimer's disease (2). In Alzheimer's disease, cerebrovascular pathology is also a frequent finding (3). Among other causes, these disease entities are considered to be associated with cerebral small vessel disease (CSVD). CSVD comprises different structural changes observed in post-mortem or *in-vivo* brain imaging, all of them related to alterations of small brain arteries. These include small subcortical infarcts, lacunes, dilated perivascular spaces, cerebral microbleeds, and particularly white matter hyperintensities of presumed vascular origin (WMH).

According to the *Standards for Reporting Vascular changes on nEuroimaging* (STRIVE)—an international consensus on the definition of cerebral small vessel disease—WMH are hyperintensities on T2-weighted magnetic resonance images (MRIs), which are located in the white matter and of varying size (4). Affecting preferentially the elderly, WMH are associated with cognitive impairment, mortality, increased risk of stroke and play a role in the development of late-onset depression (5–7). They are further considered to worsen gait (8), balance (9), and urinary function (10). Common cardiovascular risk factors associated with WMH (11), include hypertension (12), smoking (13), and diabetes (14). Nevertheless, the exact etiology and pathogenesis of WMH, as well as their role in neurodegeneration, is not fully understood. Therefore, further research on WMH is necessary to clarify these questions and guide future treatment and preventive interventions.

**Figure 1:**
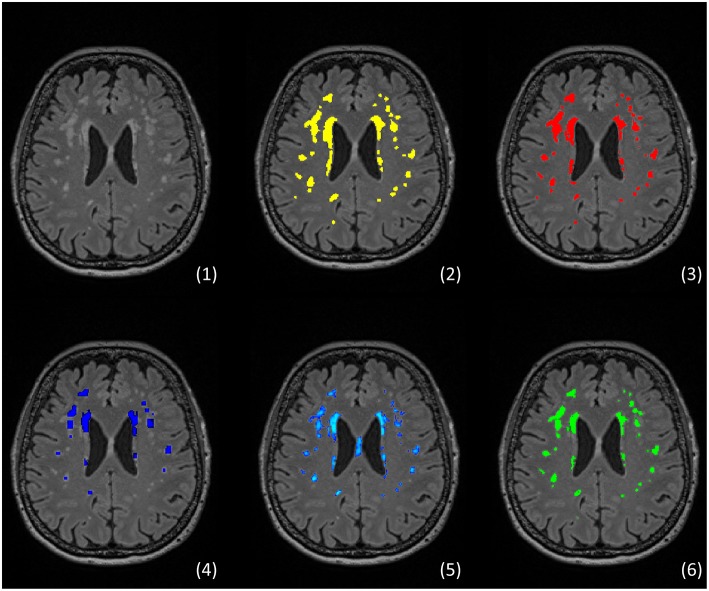
Example of segmentation of white matter hyperintensities (WMH) using different approaches The figure shows an example from an own unpublished dataset: (1) FLAIR showing typical distribution of WMH, (2) manual segmentation rater 1 (MP), (3) manual segmentation rater 2 (CM), (4) automated segmentation via Lesion growth algorithm (LGA) of LST toolbox version 2.0.15 (22), (5) automated segmentation via Lesion prediction algorithm (LPA) also of LST toolbox, (6) automated segmentation via the Brain Intensity AbNormality Classification Algorithm (BIANCA) implemented in FSL (23).

For epidemiological research, quantitative assessment of WMH is a crucial requirement for adequate analysis of associated risk factors and clinical deficits. Semi-quantitative assessments using visual rating scales (15, 16) carry certain disadvantages such as limited accuracy, high intra- and inter-rater-variation (17), low comparability (18), and inadequate depiction of longitudinal changes (19). Moreover, visual rating scales usually do not reflect precise localization of observed WMH. Although correlating with visual rating scales (20), quantitative measurements based on WMH segmentation offer a more reliable, sensitive, and objective alternative (21), which also enables the anatomical analysis. Technically, WMH segmentation is the process of subdividing image voxels into subgroups based on predefined features such as signal intensity. Figure 1 illustrates representative results of different segmentation techniques for exemplary purposes. Since segmenting brain lesions by hand is a highly demanding process, the vivid research field produced various automated and semi-automated segmentation techniques (24). Nevertheless, there are no standardized approaches to quantitative or semi-quantitative WMH segmentations. Also, inconsistent definitions of WMH (4) and differing standards for the qualitative evaluation and quantitative comparison of the results to a so-called gold standard exist, not to mention the reporting of these. The research community has recognized these problems and addressed them over the last years, with the STRIVE as a major milestone achieved in 2013: in this position paper, experts in the field provided an unification of cerebral small vessel disease definitions including a clear definition of white matter hyperintensities of presumed vascular origin (4).

Currently, there is accumulating evidence pointing to a clinical relevance of WMH, substantially driven by large-scale studies. Thus, standardization of methodological approaches for WMH characterization in these studies is of crucial importance. In this systematic review, we provide an overview of large-scale studies assessing WMH quantitatively over the past 14 years. We describe their characteristics, research subjects, approaches on WMH segmentation, and the study-specific and general development of segmentation techniques. Furthermore, we continue the discussion about the heterogeneity issues in this particular field of research. By this, we aim to contribute to the unification work of the field started previously by other research groups.

## Methods

We conducted a systematic review according to the Preferred Reporting Items for Systematic Reviews and Meta-Analysis (PRISMA) Statement (25). The review protocol was not registered in advance, the completed PRISMA checklist can be found in the Supplementary Material.

### Search Strategy and Study Selection

The methods of study selection, including searched data sources and selection criteria, were determined in advance. Two reviewers (BF, MP) carried out the literature research in December 2018 by searching the online-database Pubmed for eligible records. Search terms and applied filters are presented in the Supplementary Material.

Study selection was performed by both reviewers independently by screening abstracts or if necessary full-text papers for exclusion criteria. Exclusion criteria were specified as follows: (1) sample size <500, (2) a publication date earlier than 01.01.2005, (3) age <18 years, (4) written in another language than English, (5) no WMH segmentation has been performed, (6) review articles, (7) investigation of WMH of non-vascular origin (studies on WMH occurring in inflammatory or neurodegenerative conditions like multiple sclerosis, lupus, Sneddon syndrome, Huntington-like diseases, neurofibromatosis, leukodystrophies, cerebral autosomal dominant arteriopathy with subcortical infarcts and leukoencephalopathy, Fabry disease, sickle cell disease, progressive multifocal leukoencephalopathy, cerebral amyloid angiopathy, posterior leukoencephalopathy syndrome). Studies were included if no exclusion criteria were met.

### Data Extraction and Analysis

Data extraction was conducted independently by both reviewers reading the full-text articles. Resulting data were cross-checked afterwards. Extracted information included the name of the population study the articles belong to, year of publication, sample size, mean age of subjects in the study, the cohort included, and segmentation details like the definition of WMH, the segmentation method, reference to methods papers as well as validation measurements. Additionally, referenced methods papers were surveyed for further details on segmentation methods. All descriptive results are given by the mean ± the standard error of the mean. Data that was not available is reported as missing as long as there was no possibility to compute it.

In accordance with previous work in this field, the methods underlying the image segmentation were categorized into manual, semi-automated, and automated (24). A method was considered “manual” if the researcher annotates all lesion voxels himself; “semi-automated,” if the researcher intervenes in certain situations and “automated,” if there is no necessity of human intervention in the computing process. The latter was again classified in supervised and unsupervised depending on whether or not the classification algorithm requires a previously produced reference segmentation dataset, defining the affiliation of voxels to a particular group, e.g., WMH or non-WMH.

Furthermore, papers were characterized by the type of the underlying research question related to WMH, i.e., whether they studied the association of risk factors and WMH, the influence of WMH on a certain pathology, both directions of causation, or neither of them. All research subjects (e.g*., IL-6* or *CRP*) were extracted and assigned to subcategories defined by umbrella terms (e.g., *Inflammatory markers*). Since age and sex are regularly control variables, they are not mentioned as distinct research subjects.

## Results

### Search Results

**Figure 2:**
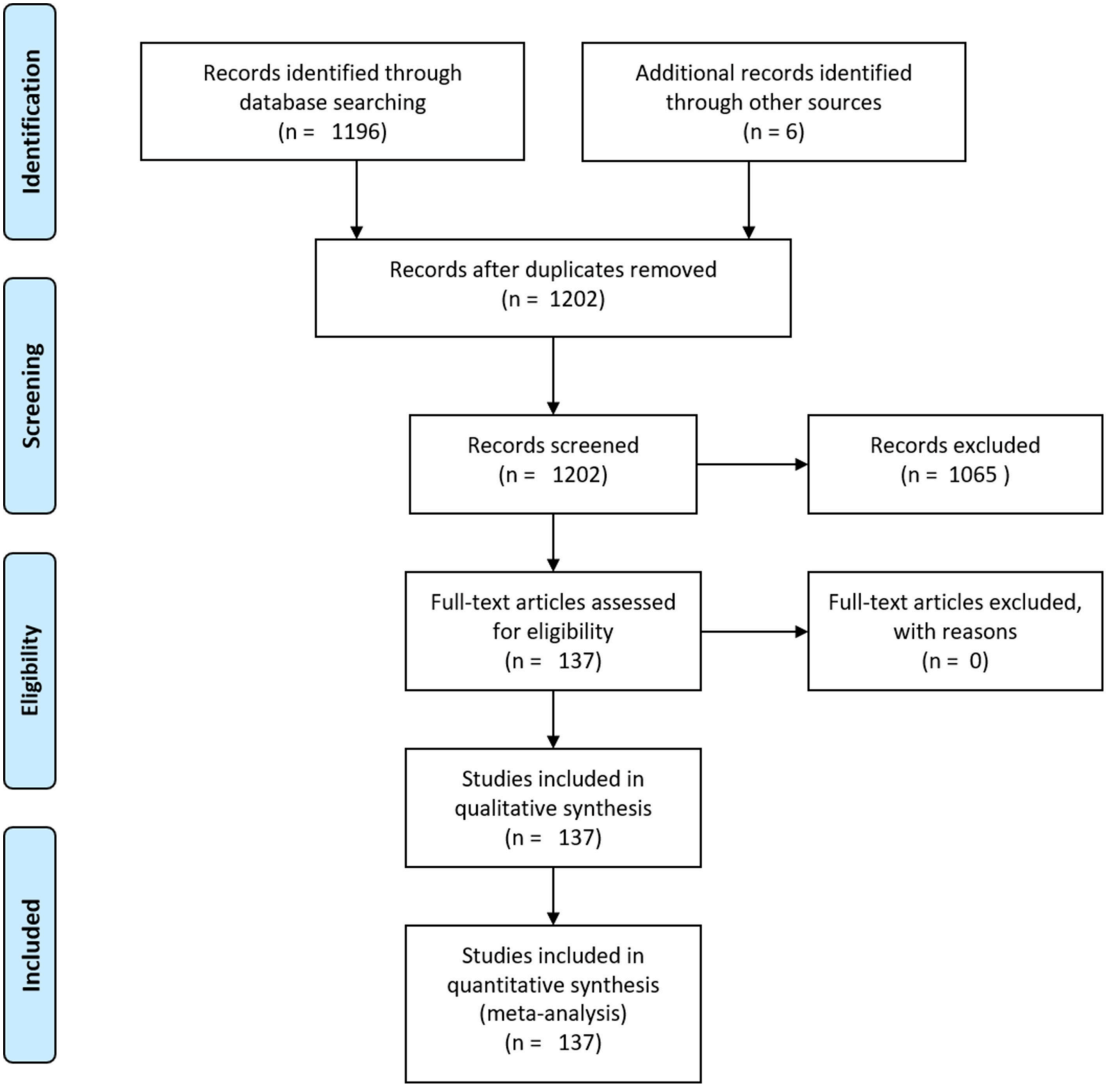
Flowchart of the search and selection process: the Pubmed research yielded 1,196 articles at baseline. No other sources for article identification were used. After application of exclusion criteria, 137 articles remained.

A flowchart summarizing the search and selection process is provided in Figure 2. Applying the aforementioned search terms and filters, the PubMed search yielded 1,196 potentially relevant records. We ruled out 1,065 of them as they met the exclusion criteria. Six further articles classified as relevant were added manually. A total of 137 articles fitting the criteria remained and were included in this systematic review. An overview of the six studies with most included articles is also part of the results section, encompassing study characteristics and their segmentation approach.

### Study Characteristics

**Figure 3:**
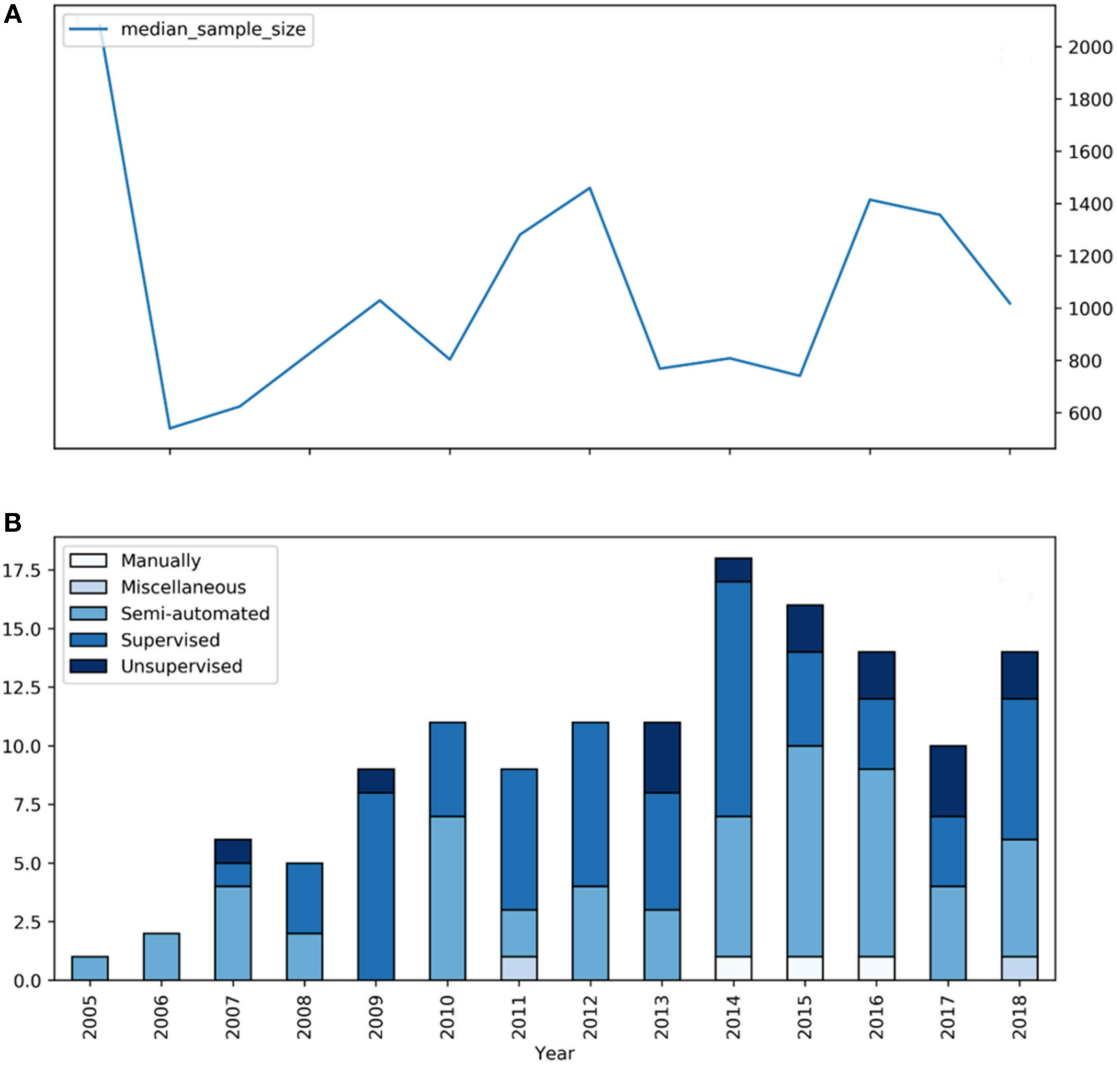
Segmentation types and mean sample size of studies on WMH between 2005 and 2018. **(A)** The blue graph represents the median sample size of the according studies. **(B)** The blue bars represent the number of large-scale studies for each year included in our review with the specific segmentation type.

The main characteristics of the studies incorporated in this review are shown in Table 1. 137 articles were included, whereas 134 belonged to 37 large-scale prospective cohort studies Box 1 delineates the 5 cohort studies that contributed the most articles to this review. The median sample size was 1,030, ranging from 501 to 9,361. Mean study sample size did not increase over the 14 years investigated (Figure 3). The mean age of subjects in the studies ranged from 46 to 83 years with a total mean of 67 ± 0.8 years. Regarding sample characteristics, 88 of the 137 studies described investigations in a standard population, while 32 included patients with a specific pathology. Seventeen studies compared their pathological cohort with a healthy control group. Concerning the underlying research question, 80 studies analyzed the relationship of risk factors and WMH, which could be categorized into 50 different thematic groups (Table 2). Sixty-four studies examined the link of WMH to diseases and vice versa, covering 25 different thematic groups (Table 3). Two papers did not fit this way of categorization. Their research subjects were “White matter hyperintensities and normal-appearing white matter integrity in the aging brain” (121) and “Incidental Findings on MRI” (122). Two studies, the Leukoaraiosis And DISability Study (LADIS) and the Genetics of Microangiopathic Brain Injury (GMBI) study, were originally established especially for research in WMH and their associations, not for other or more general topics.

**Table 1:** Characteristics of large-scale study-samples incorporated in this review.

**Cohort study**	**Incorporated articles**	**Years published**	**Mean sample size**	**Mean age**	**Sample**	**Segmentation method**	**Gold standard**	**Methods paper**
3C	15	2008–2017	1493	72	HS	Bayesian classifier, supervised, intensity thresholding, semi-automated	Visual rating scales, none	(26, 27)
ADNI	3	2010–2015	752	75	MS: MCI/AD	Markov random field, semi-automated	Semi-automated segmentation	(28)
AGES-Reykjavik	7	2009–2015	3975	76	HS	Artificial neural network, supervised	Manual segmentation	(29)
ARIC MRI	4	2013–2016	1193	65	HS	Intensity thresholding, unsupervised	Manual segmentation	(30)
ARIC-NCS	1	2017	1713	75	MS: Atherosclerosis Risk	Intensity thresholding, unsupervised	Manual segmentation	(30)
ASPS	1	2016	762	65	HS	Intensity thresholding, semi-automated	None	None
ASPS/ASPFS	1	2014	584	67	HS	Region growing, semi-automated	None	None
CDOT	1	2013	713	70	MS: DM II	Watershed transformation, unsupervised	Manual segmentation	(31)
CHAP	2	2010-2014	573	80	MS: dementia	Intensity thresholding, semi-automated	Manual segmentation	(32, 33)
CHARGE	1	2011	9361	70	MS:	Miscellaneous	None	None
EVA	1	2011	780	69	HS	Bayesian classifier, supervised	Visual rating scales	(27)
FHS	1	2017	1527	60	HS	Intensity thresholding, semi-automated	Manual segmentation	(32, 33)
FOS	13	2007–2018	1398	62	HS	Intensity thresholding, semi-automated	Manual segmentation	(32, 33)
FOS/FHS	1	2005	2081	62	HS	Intensity thresholding, semi-automated	Manual segmentation	(32, 33)
GEN III	1	2016	1995	46	HS	Intensity thresholding, semi-automated	Manual segmentation	(32, 33)
GeneSTAR	2	2014–2015	654	51	MS: Relatives of early onset CHD patients	Manual segmentation	None	None
GENOA/GMBI	4	2007–2017	1182	62	MS: Siblings of hypertensive patients, antihypertensive medication	Intensity thresholding, unsupervised	Manual segmentation	(30)
HUNT MRI	1	2018	862	59	HS	Manual segmentation and freesurfer	None	None
ILAS	1	2018	802	59	HS	Region growing, unsupervised	None	(22)
LADIS	5	2007–2016	594	74	PS: WMH	Region growing, semi-automated	None	(18)
LBC 1936	6	2014–2018	676	73	HS	Multispectral coloring modulation and variance identification, unsupervised	Semi-automated segmentation	(34)
MCSA	1	2016	1044	78	HS	Region growing, semi-automated	None	(35)
NACC UDS (Databank)	1	2018	694	73	MS: AD, MCI	Intensity thresholding, semi-automated	Manual segmentation	(32, 33)
No specific cohort study	3	2010–2016	1703	65	MS, PS: Stroke	Intensity thresholding, semi-automated	None	(26, 36, 37)
NOMAS	7	2011–2018	1216	70	HS	Intensity thresholding, semi-automated	Manual segmentation	(32, 33) None
PoP/Sunnybrook	1	2018	820	71	MS: AD, MCI, Dementia	Adaptive local thresholding	None	(38)
PROSPER	2	2006	541	75	PS: Vascular disease or high cardiovascular risk	Fuzzy inference system, unsupervised	None	(39)
RS	10	2007–2018	2378	62	HS	k-Nearest neighbor, supervised	Manual segmentation	(40, 41)
SHIP	1	2016	2367	52	HS	Support vector machine, supervised	Manual segmentation	(42)
SHIP/BLSA	1	2018	2143	74	HS	Not specified	None	None
SMART-MR	22	2008–2015	818	58	PS: Symptomatic atherosclerotic disease	k-Nearest neighbor, supervised	Manual segmentation	(43, 44)
SNAC-K	1	2016	501	71	HS	Manual segmentation	None	None
TASCOG/Sydney-MAS	1	2014	655	75	HS	Intensity thresholding, unsupervised	Visual rating scales	(45)
UK Biobank	2	2018	8439	62	HS, PS: WMH	k-Nearest neighbor, supervised	Manual segmentation	(23)
WHICAP	10	2008–2018	831	77	HS	Intensity thresholding, semi-automated, fuzzy inference system, unsupervised, region growing, unsupervised	Manual segmentation, Semi-automated segmentation	(46–48)
WHICAP/ESPRIT	1	2014	1233	81	HS	Region growing, unsupervised	Semi-automated segmentation	(47)
WHIMS-MRI	1	2014	729	83	HS	Support vector machine, supervised	Manual segmentation	(42)

*3C, Three-City Study; ADNI, Alzheimer's Disease Neuroimaging Initiative, AGES-Reykjavik, Age, Gene/Environment Susceptibility-Reykjavik Study; ARIC, Atherosclerosis Risk in Communities; ARIC-NCS, Atherosclerosis Risk in Communities Neurocognitive Study; ASPS, Austrian Stroke Prevention Study; ASPFS, Austrian Stroke Prevention Family Study; BLSA, Baltimore Longitudinal Study of Aging; CDOT, Cognition and Diabetes in Older Tasmanians; CHAP, Chicago Health and Aging Project; CHARGE, Multiple studies in CHARGE Consortium; ESPRIT, European/Australasian Stroke Prevention in Reversible Ischemia Trial; EVA, Epidemiology of Vascular Aging; FOS, Framingham Offspring Study; FHS, Framingham Heart Study; GEN III, Third Generation Cohort; GeneSTAR, Genetic Study of Aspirin Responsiveness; GENOA, Genetic Epidemiology Network of Arteriopathy; GMBI, Genetics of Microangiopathic Brain Injury; HUNT, Nord-Trøndelag Health study; LADIS, Leukoaraiosis and DISability Study; LBC1936, Lothian Birth Cohort 1936; MCSA, Mayo Clinic Study of Aging; NACC UDS, National Alzheimer Coordinating Center databank; NOMAS, Northern Manhattan Study; PoP, proof-of-principle cohort; PROSPER, PROspective Study of Pravastatine in the Elderly at Risk of cardiovascular disease; RS, Rotterdam Study; SHIP, Study of Health in Pomerania, SMART-MR, Second Manifestations of Arterial Disease-Magnetic Resonance; SNAC-K, Swedish National study on Aging and Care in Kungsholmen; Sunnybrook, Sunnybrook Dementia study; Sydney-MAS, Sydney Memory and aging study; TASCOG, Tasmanian Study of Cognition and Gait; Sydney MAS, Sydney Memory and Aging Study; WHICAP, Washington Heights-Hamilton Heights-Inwood Community Aging Project; WHIMS, Women's Health Initiative Memory Study; mean age in years; SP, Standard Population; MS, Mixed Sample; PS, Patient Sample; CHD, Coronary heart Disease*.

Box 1 **The Big 5: Cohort studies with the most contributing articles in this work.**

**SMART-MR**

With 22 articles the Second Manifestations of ARTerial disease—Magnetic Resonance Study (SMART-MR) made up the biggest proportion of all included studies. Localized in the Netherlands, SMART-MR had initially been designed to investigate the brain changes on MRI in patients with symptomatic atherosclerotic disease, namely, manifest coronary artery disease, cerebrovascular disease, peripheral artery disease, and abdominal aortic aneurysm. Recruitment took place from May 2001 until December 2005 and resulted in a baseline sample size of 1,309 subjects (49, 50).

**3C**

Established in the three French cities Bordeaux, Dijon, and Montpellier, the objective of the 3C-study was the assessment of risk of dementia and cognitive impairment attributable to vascular factors. 9294 older adults form the original sample size, recruited from March 1999 to March 2001 (51).

**Framingham Offspring Cohort**

The Framingham Offspring Cohort contains the offspring of participants from the original Framingham Heart Study. Founded in requirement of a young study sample, the enrolment phase in 1971 supplied an initial study sample of 5,124. The study's purpose is described as the identification of common factors contributing to cardiovascular disease (52, 53).

**WHICAP**

The Washington/Hamilton Heights-Inwood Columbia Aging Project, located in New York, investigates in Alzheimer's Dementia and Aging in a cohort of multiple ethnicities. The original cohorts size counts 3,452 members (54).

**Rotterdam Study**

Situated in the Netherlands, the enrolment of the Rotterdam study started in 1990 with the baseline sample size of 7,983 participants. Having a broader approach, the study covers multiple diseases of elderly people in its investigations, i.e., cardiovascular, neurological, ophthalmological, endocrinological, and psychiatric diseases (55).

**Table 2:** Overview of supposed risk factors for WMH in large-scale studies.

**Risk factors**	**Studies**
Ad-genetics	**(****56****)**
Adiposity	**(****58****)**
Angiotension converting enzyme	**(****59****)**
Antihypertensive treatment	**(****60****)**
Aortic stiffness	**(****61****)**
ApoE genotype	(**148**, **178**, **182**, 74, 91, **58**)
Arterial stiffness	(**191**, 132, 171, **141**)
Atherosclerosis	(**183**, **49**, **179**)
Atrial fibrillation	(138, **163**)
Blood pressure variability	**(****62****)**
Cardiac stress markers	**(****63****)**
Cardiovascular risk factors	(143, **158**, 56)
Common risk factors	(**152**, **98**, 187, **135**, **176**, **193**, 79)
Conjougated equine estrogen	(64)
Diabetes mellitus type II	(**136**, 165, 192, **175**, 14, 153, **144**)
Diet quality	(167, **150**)
Dysglycemia	**(****65****)**
Exhaled carbon monoxide	**(****66****)**
Extracellular vesicle protein levels	**(****67****)**
FGF23 elevation	**(****68****)**
Folate	(69)
Genetic loci	**(****70**, **71****)**
Hba1C	**(****72****)**
Homocystein	(69, 72, 142, **161**)
Hyperlipidemia	(73)
Hypertension	(**75**, 182, **173**, **187**, 132, **149**, **174**, 146)
Inflammatory markers	(**115**, **85**, 159, **186**, **168**, 57)
Leisure activity	**(****74****)**
Lipoproteins	**(****75****)**
Metabolic syndrome	(76)
Metalloproteinases	**(****77****)**
Midlife obesity	(78)
Nocturnal blood pressure	(79)
Parathyroid hormon	**(****80****)**
Parental longevity	**(****81****)**
Parental stroke	**(****82****)**
Perceived stress	(83)
Physical activity	(84)
Plasma beta-amyloid	**(****85**, **86****)**
Red blood cell omega-3 fatty acid	**(****87****)**
S100B	(88)
Sleep duration	**(****89****)**
Sulfur amino acids	(69)
Thyroid function	(90)
Tomm40 523 genotype	(91)
Uric acid	(92)
VCAN snps	(93)
Vitamin B12	(69)
Vitamin D	(94)
Vo2Max	(95)

*Common risk factors are age, sex, gender, and ethnicity. Significant associations with WMH indicated in bold*.

**Table 3:** Overview of supposed sequelae of WMH in large-scale studies.

**Sequelae**	**Studies**
Alzheimer's disease	**(****96****–****99****)**
Antidepressant Use	**(****100**, **101****)**
Apathy symptoms	**(****102****)**
Brain atrophy	(**181**, **182**, **184**, 56, 172, **145**)
Brain volumetric changes	(**32**, 162, 189)
Callosum atrophy	**(****103**, **104****)**
Cerebral blood flow	**(****105****)**
Cognitive function	**(****21**, **56**, **62**, **95**, **153**, **79**, **104**, **134**, **140**, **143**, **155**, **156**, **160**, **164**, **166**, **169**, **170**, **180**, **188****–****190**, **194**, **195****)**
Death	**(****106****)**
Depressive symptoms	(100, **101**, **154**,^133^,**147**, **151**, **177**, 139)
Falls	**(****107****)**
Functional status	**(****108****)**
Grief	(109)
Headache	**(****110****–****112****)**
Immobility	**(****57****)**
Manual dexterity	**(****113****)**
Migraine	**(****110**, **112****)**
Mild cognitive impairment	(**98**, 137, **185**, **157**)
Olfactory function	(114)
Perivascular spaces	(115)
Restless-Legs-syndrome	(116)
Retinal Microvasculature	**(****117****)**
Study-drop-out	**(****118****)**
Subjective memory Impairment	**(****119****)**
Tract Integrity	**(****120****)**

*Significant associations with WMH indicated in bold*.

### Segmentation

#### Definition of White Matter Hyperintensities

Only 24 (17.5%) articles contained an explicit definition of WMH. The remaining studies either gave an implicit explanation through their segmentation method or had no specific definition of WMH. Of the studies included in our review, 72 were published since 2014, i.e., after publication of the STRIVE paper. Of these, 15 defined WMH explicitly, 10 of them according to STRIVE. Forty-seven studies did not refer to any explicit definition of WMH at all.

#### Segmentation Types and Segmentation Techniques

The largest proportion of studies applied automated segmentation techniques: supervised and unsupervised segmentation were used in 60 and 15 articles, respectively. Fifty-seven articles described a semi-automated segmentation technique, while only 3 papers relied on manual segmentation and 2 papers described a miscellaneous approach. Studies using fully automated methods had a significantly higher sample size (*p* = 0.002; Student's *t*-test) compared to semi-automated methods (mean 1017.0 vs. 1650.8). Figure 3 shows the distribution of the segmentation types over the years. The peak of published articles on WMH was in 2014. We identified 17 different segmentation techniques used in the studies included in our review (Table 1). Box 2 delivers an introductory explanation for the 5 most employed techniques.

Box 2 **Top 5 most used methods for WMH-segmentation in large-scale studies.**

**Intensity thresholding—DeCarli et al. (32, 35)**

The semi-automated method is based on the work of DeCarli et al. Taking the dataset with unclassified voxels, the examiner models a gaussian curve based on the voxel intensity values. Afterwards a threshold value of 3.5 standard deviations above the mean is set. Every voxel with an intensity value higher than the threshold value is defined as a white matter hyperintensity voxel.

**Region Growing—Brickman et al. (47)**

Similar to the approach by DeCarli, the approach by Brickman and colleagues starts with an intensity thresholding step (2.5 standard deviations) to determine seed voxels for each hemisphere. The seeds are the origin of region growing processes. Every seed voxel intensity value serves as calculation base of an interval (±5%). The algorithm determines class membership of every adjacent voxel by looking whether its intensity value falls into that interval. The algorithm moves further by considering every new WMH defined voxel as a seed with its own interval.

**k-Nearest Neighbors—Anbeek et al. (43, 44) and de Boer et al. (40)**

Utilized by the Rotterdam study as well as SMART-MR, k-NN is a supervised machine learning algorithm that aims to classify objects based on multiple features, e.g., voxel intensities in T1-w, IR, PD, T2-w, and FLAIR as well as spatial information. For every preclassified voxel a certain location in a multi-dimensional feature space is calculated. Subsequently, a probability is allocated to every voxel of unknown classification based on the labels of its k nearest neighbors in the feature space.

**Naïve Bayesian Classifier—Maillard et al. (27)**

The Naïve Bayesian Classifier is a machine learning algorithm utilizing Bayesian statistics. As a learning step a preclassified dataset is consigned. The algorithm takes this dataset and calculates its baseline probabilities: simple probabilities like the likelihood of choosing a WMH through a random pick [P(WMH)] and conditional probabilities like the likelihood of a choosing a WMH through a random pick under the assumption of certain features [P(WMH|Features)]. Next, unclassified voxels are handed over to the algorithm. Based on the baseline probabilities the algorithm delivers probability values of group membership given certain features for every voxel. Finally, those membership values are compared and the voxel is assigned to the group with the highest (24).

**Artificial Neural networks—Zijdenbos et al. (29)**

Artificial neural networks are algorithms inspired by the architecture of biological neural networks, containing neurons and in-between connections. The network established by Zijdenbos and colleagues consists of three layers: an input layer counting consisting of six nodes/neurons where the spatial and intensity information is handed over to the algorithm, a hidden layer with 10 nodes that processes information the input layer delivers and an output layer with two nodes determining the classification of non-WMH and WMH.

### Validation Methods

Methodological validation was done by application of accuracy and reproducibility measurements. Of 60 articles with semi-automated or manual segmentation techniques, 18 (30.0%) validated their results with reproducibility metrics, namely the intraclass-correlation coefficient and intra-rater repeatability. Of 132 articles using semi-automated and automated segmentation techniques, 112 (84.8%) reported accuracy metrics like Dice similarity index, intraclass-correlation coefficient, mean absolute error, Pearson's correlation, Cronbach's alpha, Spearman's correlation coefficient, ANOVA, and ANCOVA to validate their results. The gold standard the segmentation techniques were tested against was manual segmentation in 84 studies, while 16 and 13 tested against visual rating scales and semi-automated techniques, respectively.

## Discussion

In this systematic review, we identified 137 papers from large-scale studies applying a quantitative analysis of WMH over the past 14 years. With 134 of these being part of a longitudinal prospective cohort study, this indicates to the relevance of these studies in this particular field of research. The large number of studies included in this review reflects the current scientific relevance of WMH in cerebrovascular research. The sample size of these studies ranged from 501 to more than 9,000, which demonstrates the feasibility of WMH segmentation in large samples resulting from the scalability of largely automated image analysis techniques. However, although the past years have brought ongoing improvements in automated image analysis techniques, we did not observe a clear increase of sample size over time. This may either reflect the typical delay until new analysis methods are implemented in large epidemiological studies, which usually are running over a long period. This may also be explained by other factors limiting sample size in large-scale studies beyond factors related to image analysis, e.g., recruitment, or limited capacity of study centers for clinical or imaging studies. Mean age of study subjects across all studies was 67 years, which is likely due to the fact of cerebrovascular diseases being aggregated primarily in the elderly.

The research questions addressed in the studies included in our review could be divided into two groups: the association of risk factors with WMH and supposed clinical or other consequences of WMH. The five most frequently investigated risk factors studied with regards to their association with WMH were hypertension, common risk factors, diabetes, ApoE genotype and inflammatory markers. The majority represents risk factors or markers of atherosclerosis (123).

With regard to clinical manifestations of WMH, there were two areas of interest in the focus of the reviewed studies: a large number of studies looked at WMH in the context of cognitive decline, mild cognitive impairment, or brain volumetric changes and brain atrophy, which are considered as biomarkers of neurodegeneration. This research focus appears obvious, as cerebral small vessel disease is a known risk factor for vascular cognitive impairment and vascular dementia (3). Depressive symptoms were the second clinical focus, as well-thematized in multiple studies. This is in line with the vascular depression hypothesis which proposes an association between the disruption of frontostriatal pathways by WMH and late-life depression (124, 125).

The lack of studies addressing e.g., the association of WMH and ischemic stroke and intracerebral hemorrhage (37, 126) might represent a bias in our search criteria.

Our review focused on the methods utilized for WMH characterization. To some parts, the heterogeneity and lack of standardization seem not only to be a problem of imaging analysis but also of the definition and nomenclature of findings related to cerebral small vessel disease. In an analysis of 1,144 studies dealing with WMH research, 275 used a variant term to “white matter hyperintensity” in their titles or abstracts (4). Efforts to overcome this lack of consensus on terminology and definition of white matter hyperintensities led to publishing the STRIVE consensus criteria in 2013, defining standards for research into cerebral small vessel disease (4). We also wanted to see, whether this initiative and publication of research standards had an impact on scientific studies of WMH in large cohorts. Still, a lot of unifying potential remains here, harboring the problem of arbitrary WMH segmentation and contributing another aspect to the discussion. These numbers suggest that there is still much room for the unification of scientific standards in this research area. In line with this, a recent contribution to the discussion suggested that the descriptive nature of most definitions of white matter hyperintensities is accountable for low-quality segmentation (127). The authors propose a statistical definition as a solution due to its better measurability and provide competitive results with it.

Virtually all studies relied on either semi-automated or fully-automated techniques for WMH segmentation. This finding reflects the trend toward segmentation automation resulting from the acknowledgment of limitations of manual segmentation: it is laborious, thus expensive; is prone to errors; subjective and shows high intra-rater and inter-rater variability (36). Since semi-automated segmentation techniques succumb automated ones regarding human intervention while showing similar segmentation quality, a further trend from semi-automated segmentation methods to fully automated techniques was assumable. Although automated segmentation techniques constituted the largest proportion over the past 14 years from observation of the time course of our data a clear trend toward automated segmentation was not derivable. The significantly higher sample size of studies using automated methods compared to studies using semi-automated methods can be explained by the fact that with higher sample size approaches requiring interaction with a human observer become less feasible.

One striking result of our review is the manifoldness of segmentation techniques used. Almost every cohort study identified had its own segmentation approach. Our review was not designed to answer the question, whether any of the segmentation methods is superior for WMH segmentation. Due to the inherent complexity of the segmentation task, the research field's demand for one proper automated segmentation technique remains unresolved. However, the diversity of segmentation approaches used in large-scale studies is remarkable, which in turn reflect the total lack of any consensus or agreed methodological standard for WMH segmentation.

The existence of a large variety of segmentation techniques is not inherently harmful to the field of research, as it may also be interpreted as a reflection of its vividness. However, the multiplicity of methods used for segmentation and quantification of WMH represents a scientific problem, because it leads to potential incoherence and incomparability between studies. Crucial results such as the overall WMH extent may differ in significant ways depending on the methods used for WMH segmentation.

As a relevant example of how to address cross-study heterogeneity, the NeuroCHARGE Consortium (70) used results of 7 different large-scale prospective cohort studies for a genome-wide association study (GWAS). Before conducting their analysis, they assessed the results for comparability, encompassing WMH segmentation and visual rating scale data, by examining their quality individually via comparison with a reference standard. In addition, utilized visual scoring and volumetric methods were performed on standard image data sets to test agreement.

Automated segmentation was primarily based on machine learning algorithms: for instance, k-nearest neighbors, naive Bayesian classifiers, artificial neural networks and support vector machines were successfully employed to serve the problem of quantitative WMH delineation. Since deep learning, namely convolutional neural networks (CNN), proved themselves for computer vision tasks they are also a hot contender in the WMH segmentation problem. First studies and the WMH segmentation challenge at MICCAI 2017 (http://wmh.isi.uu.nl/) delivered promising results (128–130).

In the publications analyzed in our review, some validated their segmentation results against a gold-standard—usually manual segmentation. This “gold standard,” however, has a lot of inherent limitations, resulting in a significant degree of subjectivity in the validation process. This, again, contributes to incomparability between different methods due to the fact they have been validated on hardly comparable gold standards. Moreover, the methods used for validation, also show some heterogeneity. Many studies use different parameters than the most common metrics like the Dice similarity index and thereby contribute to the overall heterogeneity and lead to aggravated comparison. Again, standardization might provide a solution. The study field could consent, just in the manner of the STRIVE, to specific parameters for validation measures including guidelines of subset selection for specific segmentation tasks (131).

Regardless of the already discussed problems, there are further contributors to variation in WMH quantification. In the end, the quality of the segmentation process depends strongly on the quality of the underlying MRI-images. Especially clinical scans are often very heterogeneous in terms of available MRI-sequences, manufacturer, field strength, signal-to-noise ratio, additional pathologies visible in the scan like stroke lesions or tumors, overall quality assurance protocols and sequence parameters like voxel dimensions, slice gaps, contrast and automated distortion correction. Therefore, the application of the discussed algorithms in the clinical routine might be only possible to a limited extent.

In conclusion, the vast number of large-scale studies reporting the results of segmentation and quantification of WHM reflects the fact that cerebral small vessel disease is a research topic of great interest, especially within the context of epidemiological studies or large patient cohorts. Both, risk factors associated with the presence and extent of WMH and possible behavioral or clinical sequelae are in the focus of research. Approaches to WMH segmentation used in these studies with large samples rely on semi-automated or fully automated algorithms. A multiplicity of methods is used, and clear definitions of WMH are only provided in a minority of studies, which limits comparability and reproducibility of results. New technical developments in segmentation methods may further improve automated lesion segmentation in the near future. In addition to technical advancements, there is a clear need for creating and adhering to reporting guidelines covering both definition of WMH and description of segmentation approach.

## Data Availability

The datasets generated for this study are available on request to the corresponding author.

## Author Contributions

BF, MP, and GT contributed to the conception and design of the review and to the writing of the manuscript. BF and MP performed the PubMed search and extracting of relevant studies. All authors contributed to the analysis of the results, to manuscript revision, read and approved the submitted version. GT supervised the project.

### Conflict of Interest Statement

The authors declare that the research was conducted in the absence of any commercial or financial relationships that could be construed as a potential conflict of interest.
